# Supplementary material for: Evidence for a Role of 5-HT-glutamate Co-releasing Neurons in Acute Stress Mechanisms
Source: ACS Chem Neurosci. 2024 Feb 20;15(6):1185–96. doi: 10.1021/acschemneuro.3c00758 (PMC10958520; doi:10.1021/acschemneuro.3c00758)
Supplement: Supplementary file 1 — cn3c00758_si_001.pdf [file cn3c00758_si_001.pdf]

Supporting Information

Evidence for a Role of 5-HT-glutamate Co-releasing Neurons in Acute Stress Mechanisms

L. Sophie Gullino[1], Cara Fuller[1], Poppy Dunn[1], Helen M. Collins[1], Salah El Mestikawy[2,3], Trevor Sharp[1]

[1]University Department of Pharmacology, University of Oxford, Mansfield Road, Oxford OX1 3QT, U.K.; [2]Douglas Mental Health University Institute, Department of Psychiatry, McGill University, Montreal, QC H4H 1R3, Canada; [3]Sorbonne Université, INSERM, CNRS, Neuroscience Paris Seine – Institut de Biologie Paris Seine (NPS – IBPS), 75005 Paris, France.

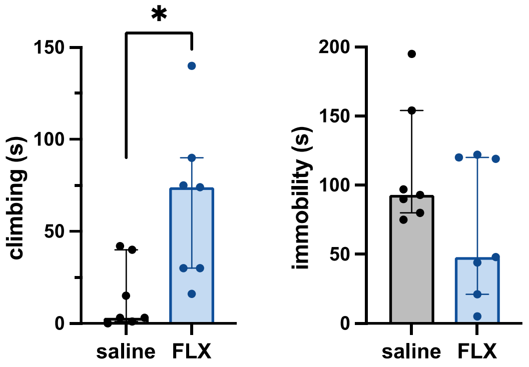

Supporting Information Figure 1 | Behavior of wildtype mice exposed to swim stress with and without fluoxetine. Time spent climbing (left) and immobile (right) during swim stress exposure. Columns represent median ± interquartile range values, with individual values indicated by closed circles. Groups were saline (n=7), and fluoxetine (FLX, n=7). *$p < 0.05$.

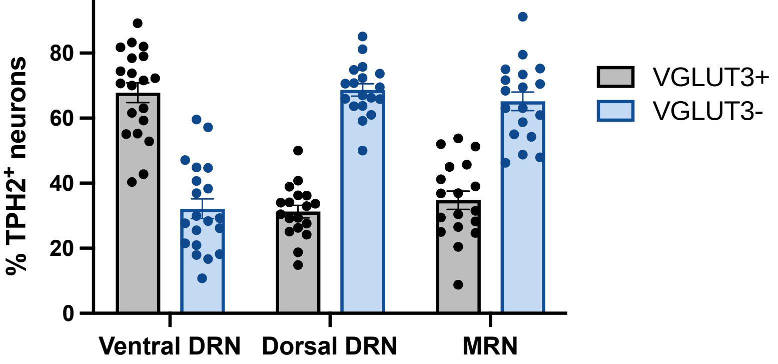

Supporting Information Figure 2 | Colocalisation of TPH2 and VGLUT3 in neurons of mouse raphe regions. Percentage of TPH2 neurons that colocalised with VGLUT3, or were VGLUT3 immunonegative (n=17-20). Bars represent mean ± SEM values, with individual values are indicated by closed circles.

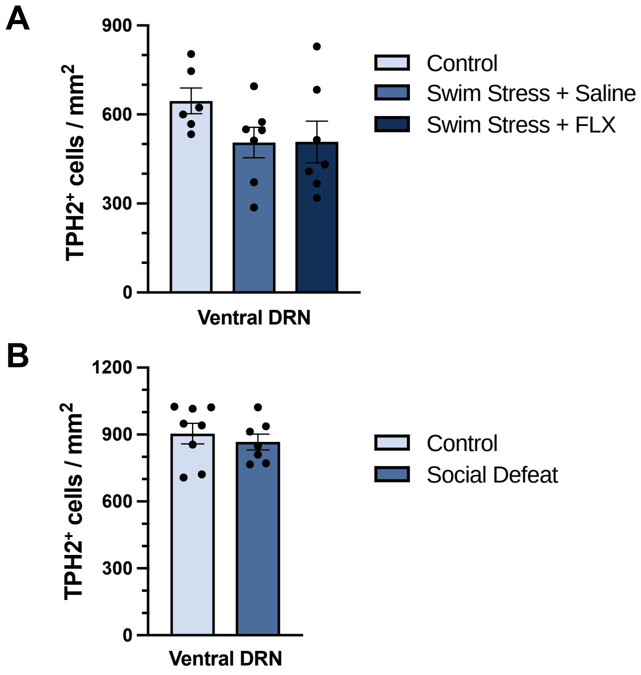

Supporting Information Figure 3 | TPH2 expression in ventral DRN of mice exposed to swim stress and social defeat. A) Number of TPH2 immunoreactive neurons following swim stress with or without fluoxetine; groups were control (n=6), saline + swim stress (n=7), and 10mg/kg fluoxetine + swim stress (n=7). (B) Number of TPH2 immunoreactive neurons following a single episode of social defeat; groups were control (n=8), and social defeat (n=7). Bars represent the mean ± SEM values, with individual values indicated by closed circles.

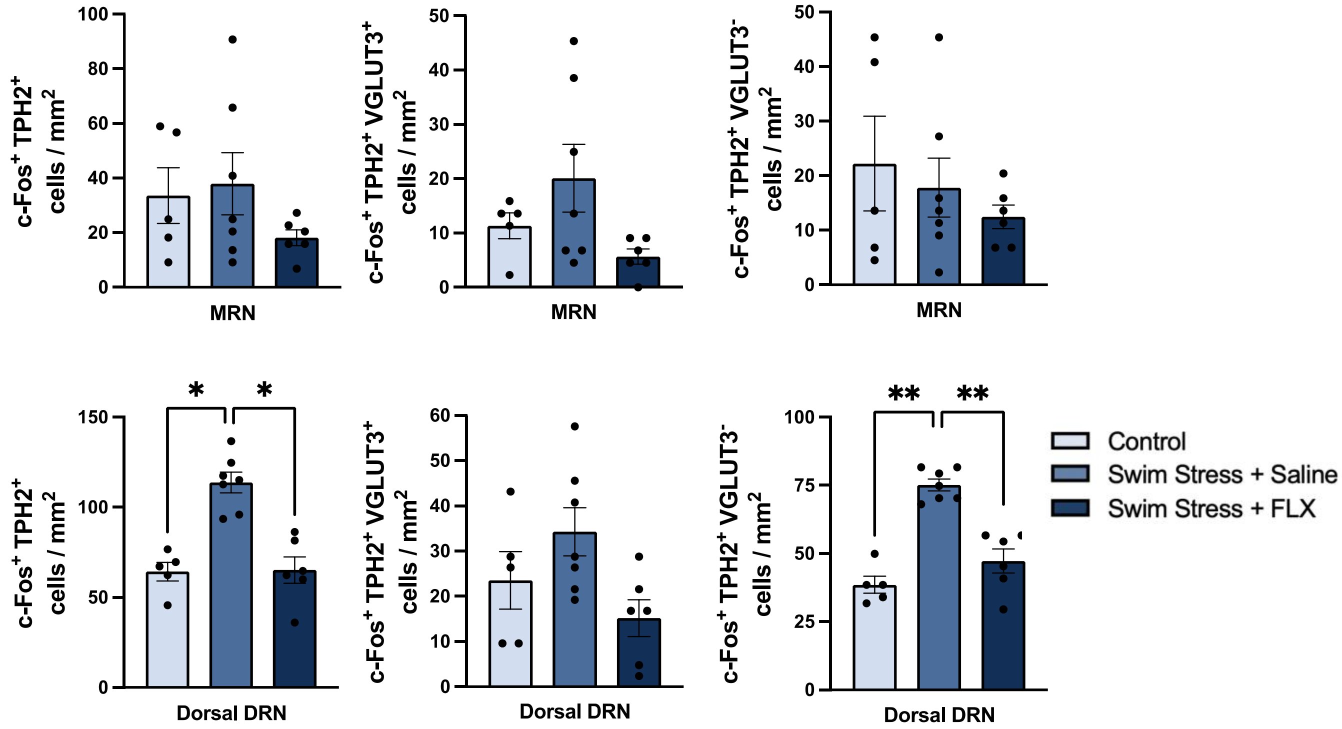

Supporting Information Figure 4 | Effect of swim stress on c-Fos expression in DRN neurons coexpressing TPH2 and VGLUT3 in the MRN and dorsal DRN. Effect of swim stress on number of c-Fos/TPH2 double-labeled neurons (left), c-Fos/TPH2/VGLUT3 triple-labeled neurons (middle), and c-Fos/TPH2 double-labeled neurons but VGLUT3 immunonegative (right). Columns represent the mean ± SEM values, with individual values indicated by closed circles. *p < 0.005 **p < 0.0001. Groups were control (n=6), saline + swim stress (n=7), and 10 mg/kg fluoxetine + swim stress (n=7). Abbreviations as in Figure 1.

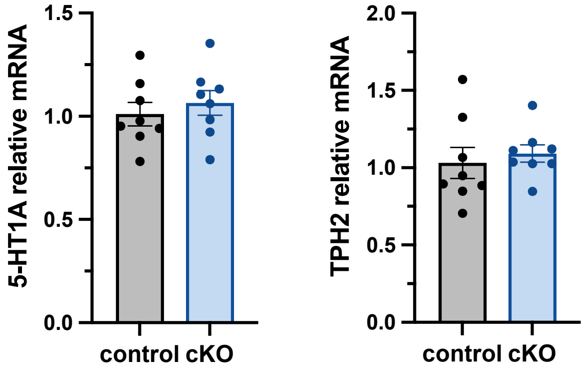

Supporting Information Figure 5 | Additional molecular characterization of the DRN of VGLUT3 cKO$^{5\text{-}HT}$ mice. TPH2 and 5-HT_{1A} mRNA in the DRN of VGLUT3 cKO$^{5\text{-}HT}$ mice, and littermate controls (n=8). Columns are mean ± SEM values, with individual values indicated by closed circles.

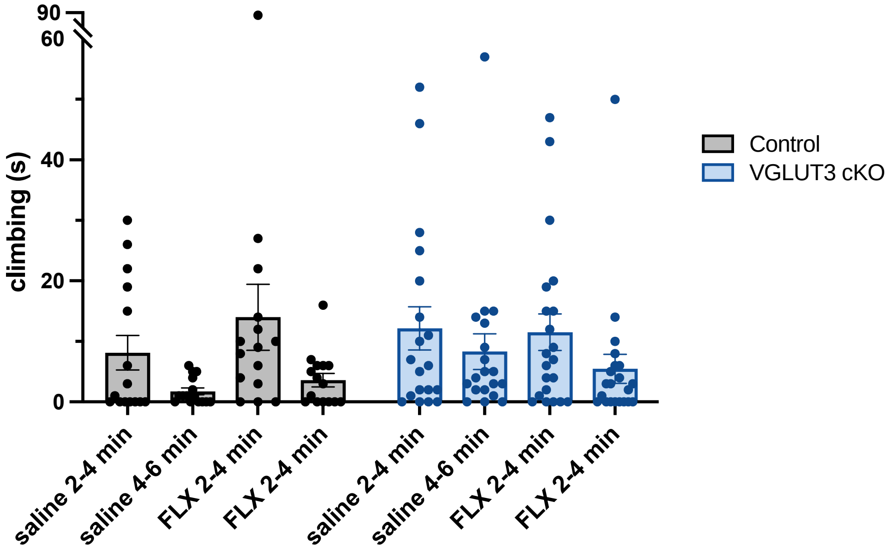

Supporting Information Figure 6 | Behavioral response of VGLUT3 cKO$^{5\text{-}HT}$ mice to swim stress in 2 min time bins. Performance of VGLUT3 cKO$^{5\text{-}HT}$ mice (n=19-20), and littermate controls (n=15) during swim stress exposure in 2 min time bins, with saline or fluoxetine (FLX). Columns are mean ± SEM values, with individual values indicated by closed circles.
